# Case Report: Ischemic stroke in a young transgender woman due to unsupervised estrogen therapy

**DOI:** 10.3389/fgwh.2025.1588553

**Published:** 2025-08-08

**Authors:** Lucas Blanco Insaurralde da Luz Silva, Andressa Camilo Oliveira, Anny Gabriela Marçal de Carvalho Araújo, Maria Luiza Ferri Cury, Isabela de Carvalho Florêncio, Amanda Boutrik, Franciely Hyun Su Barakat Kim, Egidi Mayara Firmino Silva, Luana Karen dos Santos, Renata Gratão Rezende, Rodolfo Antônio Corona, Gabriel Pereira Braga

**Affiliations:** ^1^ Faculdade de Medicina, Universidade Federal do Mato Grosso do Sul (UFMS), Campo Grande, Mato Grosso do Sul, Brazil; ^2^ Department of Internal Medicine, Santa Casa de Misericórdia de Campo Grande, Campo Grande, Mato Grosso do Sul, Brazil; ^3^ Department of Neurology, Hospital Universitário Maria Aparecida Pedrossian (HUMAP/UFMS), Campo Grande, Mato Grosso do Sul, Brazil; ^4^ Faculty of Pharmaceutical Sciences, Food and Nutrition (FACFAN), Federal University of Mato Grosso do Sul (UFMS), Campo Grande, Mato Grosso do Sul, Brazil

**Keywords:** stroke, transgender health, hormone therapy, estrogen, cerebrovascular risk, ischemic stroke

## Abstract

**Introduction:**

Stroke is often associated with the elderly population, but recent epidemiological data indicate an increasing incidence among young adults. Among the risk factors, estrogenic hormone therapy (HT) has been linked to cerebrovascular events. This report presents the case of a transgender woman who suffered an ischemic stroke during the inappropriate use of HT, highlighting the importance of medical follow-up and risk assessment in gender-affirming therapy.

**Case Description:**

A 30-year-old transgender female patient had been using estrogenic HT purchased on the black market (cyproterone 2 mg + ethinyl estradiol 0.035 mg/day) since the age of 17, without medical supervision. She had a history of HIV infection under irregular treatment and previously treated syphilis. She developed sudden-onset right-sided hemiparesis and homonymous hemianopsia. The initial computed tomography scan revealed no abnormalities, but magnetic resonance imaging showed ischemia in the territory of the left posterior cerebral artery. Etiological investigation with echocardiography, carotid and vertebral Doppler ultrasound, electrocardiogram, Holter monitoring, and thrombophilia panel revealed no abnormalities. The final etiological diagnosis was classified according to the TOAST criteria as “other causes,” attributed to the inappropriate use of estrogenic therapy. She was discharged with antiplatelet therapy, a statin, and fluoxetine, along with the discontinuation of hormone therapy and referral to transgender and vascular neurology outpatient clinics.

**Discussion:**

HT is essential in gender affirmation; however, its use is associated with increased risks of thromboembolic and cerebrovascular events. The patient in this case did not present traditional risk factors for stroke, reinforcing the suspicion of estrogen's role in the event. Studies suggest that supervised hormone therapy carries a lower risk of complications compared to indiscriminate use. Nonetheless, there are still gaps in the literature regarding the correlation between HT and stroke in transgender individuals.

**Conclusion:**

This case highlights the risks of inappropriate use of gender-affirming hormone therapy and the need for rigorous medical supervision. Given the growing access to this treatment, continuous monitoring is essential to minimize complications. Further research is needed to establish safer guidelines for the use of hormone therapy in the transgender population.

## 1 Introduction

The incidence of stroke occurs predominantly after the fifth decade of life and is considered a condition that mainly affects the elderly population (1). However, studies conducted across multiple centers and diverse populations have identified a global increase in the incidence of stroke among young adults (2–5). It is evident that the epidemiological profile of stroke has been shifting in recent decades, with a growing yearly incidence of cerebrovascular events in younger individuals.

Parallel to the rise in cerebrovascular events among young people, there has been growth in a particularly vulnerable population: transgender individuals. The transgender community has become the focus of various public health discussions, especially concerning the prevention of cerebro-cardiovascular diseases (6). In recent years, literature has indicated an increase in risks and diseases particularly associated with gender transition hormone therapy (6–10).

This report presents a case of ischemic stroke (IS) in a young transgender woman using inappropriate hormone therapy with oral contraceptives—substances often obtained from the black market, especially in countries lacking inclusive public health policies and comprehensive healthcare access (11). In such contexts, hormone use often occurs without medical prescription or professional supervision and is integrated into the gender-affirmation process (11). Therefore, this study stands out by describing an ischemic cerebrovascular event in an underreported population in the scientific literature (9): young transgender women using unsupervised combined hormone therapy. It highlights a critical gap in current scientific production (9, 10, 12), clarifies how inadequate hormone therapy use can trigger cerebrovascular events (9, 10, 12, 13), and reinforces the urgency of developing specific protocols for prevention, education, and healthcare assistance tailored to transgender individuals undergoing hormonal transition.

## 2 Clinical case

A 30-year-old transgender woman had been using estrogenic hormone therapy—obtained from the black market—without medical supervision since the age of 17 (cyproterone 2 mg + ethinyl estradiol 0.035 mg/day). She was HIV-positive (viral load of 413 on December 4, 2023, and CD4 count of 413 on October 3, 2023) and undergoing irregular treatment (dolutegravir 50 mg once daily, tenofovir + lamivudine 300 + 300 mg once daily), with a history of previously treated syphilis.

One day prior to hospitalization, the patient presented with a moderate-intensity holocranial headache. The following day, she awoke with sudden-onset right-sided hemiparesis and homonymous hemianopsia.

**Figure 1:**
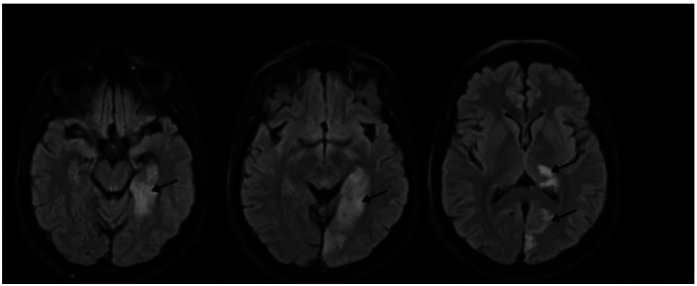
FLAIR sequence of brain magnetic resonance imaging performed on February 5, 2024, post-stroke, showing ischemia (arrow) in the territory of the left posterior cerebral artery.

Upon admission to the stroke unit, her blood pressure was 130/80 mmHg, heart rate 104 bpm, oxygen saturation (SpO₂) 99%, and she scored 5 on the National Institutes of Health Stroke Scale (NIHSS). The initial CT scan showed no signs of cerebrovascular injury, but brain magnetic resonance imaging revealed an ischemic lesion in the territory of the left posterior cerebral artery (Figure 1).

Thrombolysis was not indicated as the ictus-to-door time exceeded the therapeutic window (over 4 hours and 30 minutes). Conservative treatment for ischemic stroke was initiated, including antiplatelet therapy (acetylsalicylic acid 100 mg/day), anticoagulation (enoxaparin 40 mg/day), blood pressure control, lipid-lowering therapy (simvastatin 40 mg/day), and resumption of antiretroviral therapy (dolutegravir 50 mg/day, tenofovir + lamivudine 300 + 300 mg/day).

The patient underwent infectious screening and cerebrospinal fluid collection due to suspected neurotoxoplasmosis, neurocryptococcosis, neurotuberculosis, and neurosyphilis. Opportunistic neuroinfections were subsequently ruled out.

An etiological investigation for stroke was performed, including transthoracic and transesophageal echocardiography, carotid and vertebral Doppler ultrasound, electrocardiogram, Holter monitoring, arteriography, rheumatologic tests, thrombophilia screening, and metabolic panel—all results were unremarkable. In addition, the patient had no family history of stroke or cardiovascular disease.

Serum testosterone and estrogen levels were not measured due to the unavailability of the test in the public health system at the time of admission.

The final etiological diagnosis was classified according to the TOAST criteria as *other causes*, attributed to the inappropriate use of estrogenic therapy. After diagnosis, the patient was discharged with antiplatelet therapy (aspirin 100 mg/day), a statin (simvastatin 20 mg/day), and fluoxetine 20 mg/day. Hormone therapy was discontinued, and she was referred for follow-up at transgender and vascular neurology outpatient clinics.

The patient attended only one outpatient appointment and was subsequently lost to follow-up. During the visit, she reported adherence to her prescribed medication and discontinuation of hormone therapy. However, she continued to present with right-sided visual impairment, weakness, and thermal hypoesthesia in the right hemibody. She also reported mood changes, particularly increased aggressiveness. Her NIHSS score was 2 and her Modified Rankin Scale (mRS) score was 2. Due to the loss to follow-up, a more detailed neurological evaluation and assessment of potential complications could not be thoroughly conducted. The chronological progression of clinical manifestations, diagnostic investigations, and therapeutic decisions in the present case report is summarized in Table 1, which outlines the key events during hospitalization.

## 3 Chronological table of clinical events

**Table 1:** Chronological sequence of clinical events, neuroimaging findings, and diagnostic procedures during hospitalization.

Date, clinical event	Procedure
02/02/2024	Hospital admission at 07:10 AM. Initial symptoms: sudden right hemiparesis and homonymous hemianopsia. Initial CT scan performed: no acute findings.
05/02/2024	MRI shows ischemia in the left posterior cerebral artery territory.
07/02/2024	Transthoracic echocardiogram performed (normal results). Carotid and vertebral artery ultrasound (normal). Laboratory tests collected.
08/02/2024	Lumbar puncture: No evidence of infectious causes.
20/02/2024	Transesophageal echocardiogram performed (normal results). Rheumatologic and hematologic investigation collected. Patient discharged with aspirin (100 mg/day), simvastatin (20 mg/day), and fluoxetine (20 mg/day). Hormone therapy discontinued. Referral to neurovascular and sexuality outpatient clinics.

## 4 Discussion

A transgender individual is someone whose biological sex, assigned at birth, differs from their gender identity, which reflects how they self-identify (14). According to a study conducted at the Botucatu School of Medicine, the estimated number of transgender people currently living in Brazil is around 3 million (15). Given this figure, a deeper understanding and exploration of the specificities involved in healthcare delivery to this population has become essential. Among these specific needs are higher rates of conditions such as depression, anxiety, and sexually transmitted infections—particularly syphilis and HIV—as well as a high demand for hormone therapy for gender affirmation (14).

This latter aspect is particularly relevant when considering the increased risk of stroke in this population. Despite its significance, this remains an underexplored topic in the academic literature (9, 10, 12, 16), mainly due to the stigma associated with this population and the healthcare access barriers it faces (9, 11, 17). Hormone therapy for gender transition is one of the main pillars of gender affirmation, consisting of testosterone administration for transgender men and a combination of anti-androgens and estradiol derivatives for transgender women, aiming to develop physical characteristics aligned with their gender identity (18). Therefore, it is important to correlate the risks—particularly those related to estradiol—with the occurrence of stroke in transgender women.

The patient in this case presented with multiple potential risk factors for ischemic stroke (IS), which should be analyzed collectively. First, it is worth highlighting the prolonged use—over 12 years—of a hormonal combination (cyproterone 2 mg + ethinyl estradiol 0.035 mg/day) obtained from the black market and used without medical supervision. Although this formulation is occasionally used as a contraceptive in cisgender women, it is not recommended as standard therapy for transgender women. Self-medication without monitoring may have intensified the prothrombotic risks of estrogenic therapy, as previously reported in the literature (10, 12), especially in transgender populations lacking adequate access to healthcare.

In addition to hormone therapy, the patient was living with HIV infection, diagnosed two years earlier, and was using combination antiretroviral therapy (dolutegravir, tenofovir, lamivudine). Studies have shown that both HIV infection and antiretroviral therapy (ART) itself are associated with increased cardiovascular risk due to mechanisms such as chronic inflammation, endothelial dysfunction, lipid metabolism disorders, and a higher predisposition to atherosclerosis (19–23). Upon admission, the patient had a detectable viral load (43 copies/ml), CD4 count of 349, and CD8 count of 771, indicating moderate immunosuppression (24). Although no opportunistic or neuroinfections related to HIV were identified, her immunological status may have contributed to a baseline inflammatory state that increases the risk of ischemic events.

Regarding prior syphilis, the patient had a reactive serology (VDRL 1:16), but cerebrospinal fluid was non-reactive and showed no pleocytosis, elevated protein, or other alterations suggestive of neurosyphilis (25). Thus, a diagnosis of syphilitic meningitis or meningovascular syphilis was considered unlikely (25). From a metabolic standpoint, laboratory tests (total cholesterol, LDL, HDL, triglycerides) did not reveal dyslipidemia, and imaging studies did not show atherosclerotic plaques.

Considering the absence of cardioembolic, atherosclerotic, and direct infectious causes, the case was classified as an ischemic stroke (IS) of other determined etiology, most likely related to prolonged and unsupervised exposure to estrogenic hormone therapy, combined with the chronic inflammatory state associated with HIV infection and ART.

In this case, a critical factor supporting the hypothesis that the etiology of the IS was primarily caused by estrogenic and anti-androgenic hormone therapy is the absence of conventional risk factors such as smoking, alcohol consumption, substance abuse, obesity, hypertension, dyslipidemia, diabetes, heart disease, previous cerebrovascular events, family history of cardio-cerebrovascular diseases, and neuroinfections. Therefore, it is likely that prolonged use of hormone therapy—particularly estrogens—was associated with the ischemic event (13).

The risk of ischemic stroke and thromboembolic events associated with hormone therapy in transgender women is related to the duration of exposure, dosage, and route of administration, with patterns indicating a progressive increase in risk after two to six years of continuous estrogen use, as demonstrated by longitudinal observational studies (26).

Ethinyl estradiol is a substance capable of inducing alterations at various cardiovascular levels, which explains the high risk of thromboembolic events associated with this hormone therapy (1, 12, 27). These dysfunctions include increased thrombin and other thrombogenic factors; endothelial alterations, which predispose to atherosclerosis (linked to IS) and arterial hypertension (linked to hemorrhagic stroke risk) (28); and a reduction in HDL levels with an increase in LDL levels, contributing to the formation of atherosclerotic plaques (18).

A case-control study demonstrated a higher prevalence of transient ischemic attacks and cerebrovascular diseases in individuals undergoing gender-affirming hormone therapy (6). Similarly, a cohort study reported higher rates of ischemic stroke in transgender women, showing a tenfold increased risk compared to cisgender men (26). Moreover, cardiovascular diseases are the second leading cause of death among transgender individuals undergoing hormone therapy, ranking below suicide, which remains the leading cause of mortality in this population (8).

A recent meta-analysis sought to determine a correlation between cerebrovascular events and the use of hormone therapy in male-to-female transgender individuals (9). Ignacio et al. referenced a cohort study indicating that transgender women were more likely to develop cerebrovascular diseases (26). These findings are supported by data from a Dutch study, which identified a higher incidence rate of stroke among transgender women undergoing hormone replacement therapy compared to cisgender women [standardized incidence ratio, 2.42 (95% CI, 1.65–3.42)] (29). However, despite the presence of other studies cited in this meta-analysis that suggest an increased risk of ischemic stroke associated with hormone therapy, the estimated lifetime stroke risk in transgender women remained significantly lower than that of the general population (2% vs. 24.5%) (9). Therefore, a definitive association between gender-affirming hormone therapy and stroke cannot be established based on current evidence, given the small sample sizes, heterogeneity in routes of administration and dosages, use of convenience sampling methods, and the limited number of studies addressing this relationship (9).

When comparing hormone therapy in transgender women to that in postmenopausal cisgender women, both groups present an increased risk of vascular and cardiovascular events; however, a higher rate of cardiovascular and cerebrovascular mortality has been observed in transgender women compared to the general population (9). Current studies suggest that estrogen doses administered to transgender women should be titrated to achieve mean serum estradiol levels similar to those of postmenopausal cisgender women (10). The Endocrine Society emphasizes the importance of achieving physiologic hormone levels when comparing transgender and postmenopausal cisgender women, and specifically contraindicates the use of ethinyl estradiol for hormone therapy, due to its significantly elevated vascular and ischemic risk profile (27).

Therefore, although several studies suggest a correlation between cerebrovascular and cardiovascular diseases and gender-affirming hormone therapy, this association is not yet clearly defined in the scientific literature (9, 13, 30). The *2024 Guideline for the Primary Prevention of Stroke* currently provides specific recommendations for this population, underscoring the importance of controlling additional stroke risk factors to reduce the likelihood of cerebrovascular events, even while undergoing hormone therapy (30). Nonetheless, the guideline also highlights the existing gap in knowledge regarding stroke risk in this community (30).

The use of transdermal hormone therapy, management of metabolic and endocrine profiles (based on physiologic levels), and multidisciplinary follow-up has been associated with a reduction in cerebro-cardiovascular events in the transgender population (31, 32), representing one of the key strategies to be addressed in the clinical management of these patients.

This case report presents some limitations. The loss to follow-up prevented the assessment of medication adherence, potential long-term complications, and a more detailed evaluation of the patient's neurological recovery. The absence of serum testosterone and estrogen measurements hinders comparison with cisgender men and women of the same age group. Another limitation is the lack of biomarkers (such as IGF-1, vitamin D, and microRNAs), which could assist in understanding the relationship between ischemic stroke and estrogenic therapy. Although some studies have explored this correlation in cisgender women (33), there is still insufficient robust data to extrapolate these conclusions to the transgender population undergoing gender-affirming hormone therapy.

Furthermore, as this is a case report, the findings do not allow for generalization and may be subject to selection and information bias. Studies with greater methodological rigor, such as cohort studies and clinical trials, are necessary to confirm the association between estrogenic hormone therapy and stroke risk in transgender women.

## 5 Conclusion

This case draws attention to the increased risk of stroke in transgender individuals exposed to inadequate hormone therapy without medical supervision. As access to gender-affirming hormone therapy continues to expand, it is essential to strengthen research on the risks and preventive strategies for cerebrovascular diseases in this population.

Healthcare system avoidance among transgender individuals is often driven by factors such as discrimination, lack of inclusive care, fear of hormone therapy being discontinued, transportation difficulties, and bureaucratic barriers within regulatory systems that hinder return to specialized outpatient care. These challenges contribute to self-medication and discontinuation of clinical follow-up (11).

To address this scenario, it is necessary to strengthen specialized transgender health clinics, establish mobile outreach campaigns, train primary care teams, and revise regulatory pathways that restrict access to continuous and safe care.

Finally, cohort studies and population-based research assessing the cerebrovascular health of transgender and gender-diverse individuals are critically important. Such research should take into account factors such as duration, dose, route of administration of hormone therapy, and social determinants of health. This approach is essential for identifying risk mechanisms and guiding effective interventions to reduce ischemic events in this population (30).

## 6 Patient perspective

The patient reported satisfaction with the care received during hospitalization, highlighting the attention, support, and rehabilitation provided by the healthcare team. After discharge, she was referred to the vascular neurology outpatient clinic located within the same hospital complex. She attended a single appointment but was subsequently lost to follow-up.

In later contact with the present study’s team, the patient expressed interest in resuming outpatient care, as did her family members, showing hope regarding the continuation of specialized follow-up. In response to this request, detailed guidance and the necessary documentation were provided so that she could reenter the referral system and resume treatment at the specialized service. During this contact, she reported no additional complications or adverse events beyond those noted in her last appointment.

## Data Availability

The datasets presented in this article are not readily available because of ethical and privacy restrictions. Requests to access the datasets should be directed to the corresponding author.
